# Rapid Point-of-Care Quantification of Urinary Tract Infection Biomarker Xanthine Oxidase

**DOI:** 10.1021/acsomega.6c10153

**Published:** 2026-10-28

**Authors:** Ellen Stadler, Mark Sutton, Colin Brown, Wubbo de Boer, Rahaf Ammouri, Alison Holmes, Timothy M. Rawson, Danny O’Hare

**Affiliations:** † Centre for Antimicrobial Optimisation, 4615 Imperial College London, W12 0NN London, United Kingdom; ‡ Department of Infectious Diseases, 4615 Imperial College London, SW7 2AZ London, United Kingdom; § National Institute for Health Research, Health Protection Research Unit in Healthcare Associated Infections and Antimicrobial Resistance, 4615 Imperial College London, SW7 2AZ London, United Kingdom; ∥ David Price Evans Infectious Diseases & Global Health Group, The University of Liverpool, L69 7BE Liverpool, United Kingdom; ⊥ Fleming Institute, 4615 Imperial College London, SW7 2AZ London, United Kingdom; # Department of Bioengineering, 4615 Imperial College London, London SW7 2AZ, United Kingdom; ∇ Antimicrobial Discovery, Development and Diagnostics (AD3) UK Health Security Agency, Porton Down SP4 0JG, Salisbury, United Kingdom; ○ Institute of Pharmaceutical Science, King’s College London, London WC2R 2LS, United Kingdom; ◆ Healthcare Associated Infections, Fungal, Antimicrobial Resistance, Antimicrobial Use, and Sepsis Division, 371011 UK Health Security Agency, E14 4PU London, United Kingdom

## Abstract

Antimicrobial resistance is a growing global health threat. Overuse of antimicrobial drugs is a main driver of resistance, with urinary tract infections (UTIs) a common indication for antibiotic use. To improve UTI treatment and reduce antimicrobial resistance, real-time diagnostic tools for monitoring the host immune response are needed. Xanthine oxidase (XO) is an enzyme that is elevated in UTIs and shows potential for sensitive and selective detection of infection. We developed a rapid and sensitive point-of-care electrochemical sensor for XO detection by using a simple manufacturing approach. The sensor consists of a disposable carbon electrode embedded in a capillary fill chamber designed for point-of-care sample collection. After urine collection, XO catalyzes its substrate while changing the oxidation state of a secondary electron acceptor. The magnitude and rate of this redox shift reflects XO activity, enabling quantification of XO in urine. We demonstrate minimal intersensor variability of less than 5%, resulting in a calibration with an *R* ^2^ of 0.985 across eight individual sensors. The capillary fill device was optimized to enable efficient substrate dissolution, reducing the time to result from 10 min to just 2 min. This approach enables calibration-free detection of XO in artificial and spiked human urine samples within 2 min of sample collection in both artificial urine and spiked human urine samples.

## Graphical abstract

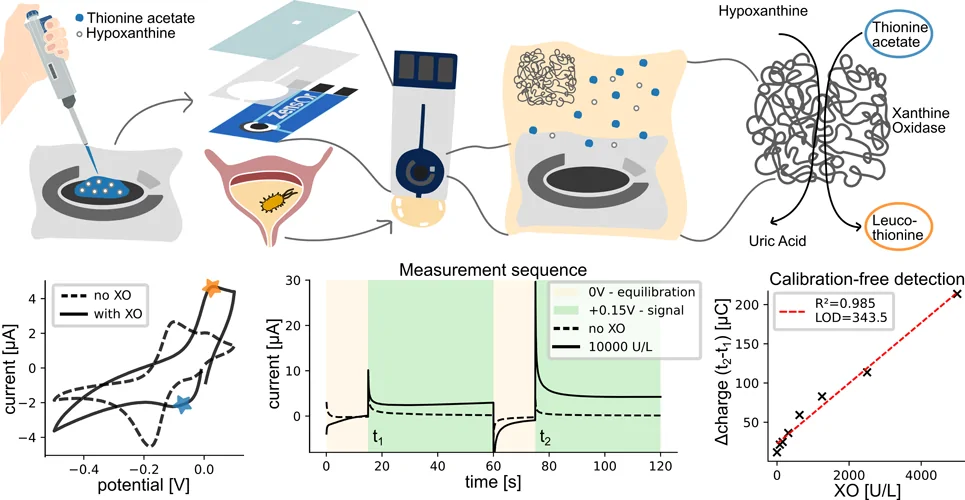

## Introduction

Antimicrobial resistance is a major global health challenge.^1^ Urinary tract infection (UTI) is a frequent indication for antimicrobial prescribing.^2^ Current antimicrobial therapies commonly use fixed dose regimens, which do not account for interpatient variability in how drugs are absorbed, distributed, metabolized, and excreted (pharmacokinetics, PK) and in the drug’s effect on the pathogen (pharmacodynamics, PD).^1^ This variability exists both across patients and within the same patient over time, highlighting the limitations of a one-size-fits-all approach to antimicrobial therapy.^1^ Traditional PK–PD targets are based on population variables and use serum drug levels and minimum inhibitory concentrations (MIC) which do not reflect drug efficacy at infection sites like the urinary tract.^3^

To optimize antimicrobial therapy and reduce the emergence of drug resistance, more targeted diagnostic tools are needed to monitor the host immune response to infection in real time. While clinical studies investigating urinary host biomarkers remain limited,^4^ recent primary research has highlighted the growing potential of direct electrochemical biosensing to measure biomarker activity directly in complex human urine matrices without pretreatment, such as the electrochemical quantification of UTI relevant biomarkers such as prostaglandin E2, interleukin-8, lipopolysaccharide,^5^ sodium nitrite,^6^ or aptamer-assisted electrochemical quantification of lactoferrin.^7^

Urinary biomarkers provide a promising avenue for developing such tools. Preliminary evidence suggests that changes in urinary biomarkers, including host-response indicators such as leukocytes, correlate with treatment efficacy.^8^ Quantitatively monitoring the bacteria-specific biomarker nitrite can distinguish between resistant and susceptible *Escherichia coli* (*E. coli*) in an in vitro model.^9^ Urinary xanthine oxidase (XO) has been identified as a highly sensitive and specific biomarker for UTI diagnosis.^10^ Elevated XO levels have been associated with positive urine culture, ranging from 17 to 271 U/L in non-UTI patients and from 5780 to 150,370U/L in patients infected with *E. coli*.^10^

Methods to detect XO described in the literature are colorimetric and fluorometric, such as the detection of XO-produced H_2_O_2_.^11^ Commercially available XO detection principles include colorimetric and fluorometric kits based on Amplex Red and ELISA kits.^12^ Colorimetric XO tests require laboratory equipment such as a spectrophotometer and involve several incubation steps with a minimal time to result of 30 min. The electrochemical detection of XO has been described as a proof-of-concept on stacked paper strips, where the deposition of hypoxanthine generated a measurable signal. The sensor has not been evaluated in artificial or human urine.^13^ Enzyme-functionalized electrochemical sensors to detect substrates have been described extensively in the literature.^14,15^ Although the redox chemistry among XO, thionine, and hypoxanthine has been described in the literature, the use of this principle to measure enzyme activity rather than substrate concentration remains unexplored. The present work does not seek to clinically validate XO as a biomarker but to develop a practical electrochemical method for its rapid quantification.

We developed an electrochemical sensor to quantify XO in urine (Figure 1). The substrate (hypoxanthine) and a redox mediator (thionine acetate) are codeposited and dried onto the working electrode Figure 1a). Adding an XO-containing sample redissolves the reagents, enabling XO to oxidize hypoxanthine and transfer electrons to the mediator. The resulting oxidation of reduced thionine acetate (leucothionine) at the electrode surface generates the analytical signal (Figure 1b).^16^

**Figure 1:**
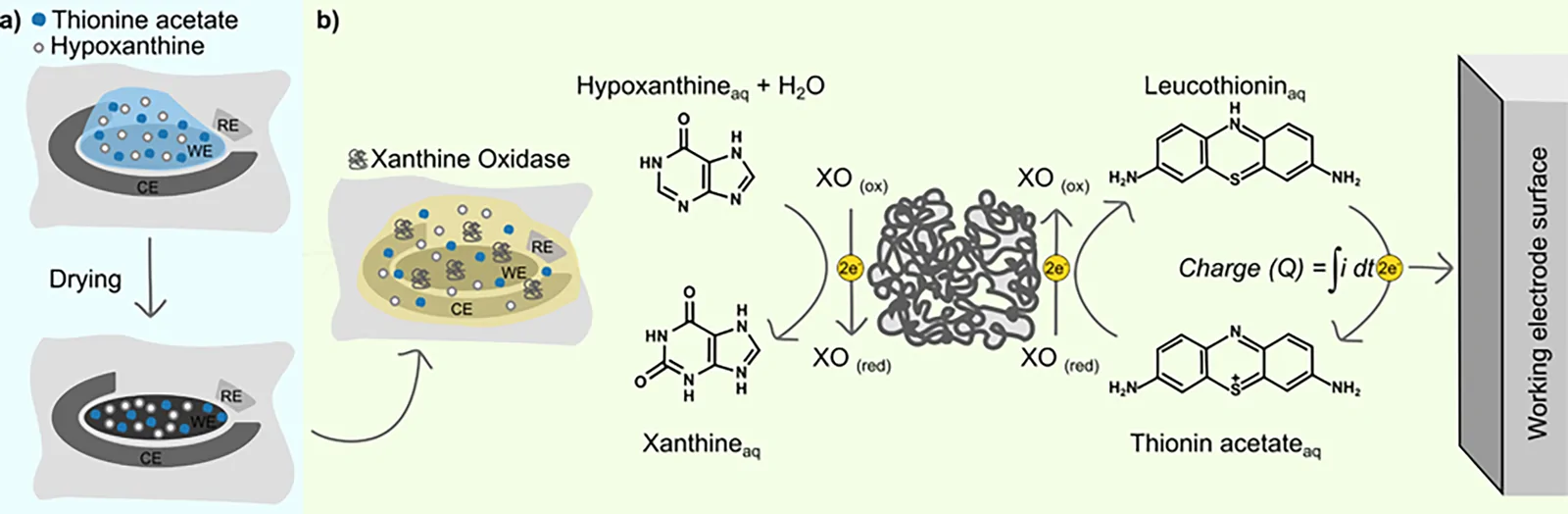
Sensor preparation and electrochemical mechanism for xanthine oxidase (XO) detection. (a) Sensor fabrication via drop-casting and drying a solution containing hypoxanthine substrate and thionine acetate mediator onto the working electrode. (b) Reaction cascade upon sample addition: XO in the urine sample dissolves the reagents and oxidizes hypoxanthine to xanthine, reducing the enzyme. Reduced XO is reoxidized by reducing thionine acetate to leucothionine. Subsequent electrochemical reoxidation of leucothionine to thionine acetate at the electrode surface generates a measurable signal proportional to XO activity.

Thionine as electron transfer mediator was selected for its kinetics with XO and for the required redox potential.^16,17^ To minimize the risk of interference thionine’s reduced form, leucothionine, is oxidized at +50 mV (vs Ag/AgCl) as shown previously,^16^ a potential where other redox active sample components such as ascorbate, uric acid, or creatinine and drugs such as paracetamol are inert.^18–19,20^ The total charge passing at the electrode when XO is interacting with hypoxanthine and thionine in the capillary fill chamber therefore correlates with XO concentration. The electrochemical behavior of thionine as transfer mediator was assessed on glassy carbon electrodes under oxygen-free conditions in absence and presence of XO using cyclic voltammetry (CV). The incubation of XO-containing sample with substrate and secondary electron acceptor was optimized to achieve highest sensitivity and lowest intersensor variability.

While previous clinical studies have identified urinary XO as a promising biomarker associated with culture-positive UTI^7^ as summarized in Supporting Information, Figure 1, the aim of the present study was not to clinically validate XO for UTI diagnosis, but rather to develop and analytically evaluate a rapid electrochemical method for XO detection in urine. Such a sensing platform could support future studies investigating XO-guided infection monitoring and antimicrobial-treatment optimization.

## Experimental Section

### Materials

Sodium sulfate, uric acid, sodium citrate, creatinine, urea, potassium chloride, calcium chloride, ammonium chloride, potassium oxalate monohydrate, sodium dihydrogen phosphate dihydrate, sodium phosphate disbasic dihydrate, HEPES, hypoxanthine, xanthine oxidase (lypophilized powder), sodium hydroxide, hydrochloric acid (37%), thionine, methylene blue, phosphate buffered saline, and trehalose were obtained from Sigma-Aldrich. Artificial urine was prepared weekly following the protocol by Sarigul et al.^21^ and sterilized using a syringe filter (Corning, nylon membrane, diameter 25 mm and pore size 0.2 μm). Xanthine oxidase activity assay kit (A319756) was purchased from antibodies.com.^22^ Then 3 mm diameter glassy carbon rod electrodes, Ag|AgCl reference electrodes (3 M NaCl aqueous solution), and platinum counter electrodes were obtained from CH Instruments. Disposable glassy carbon electrodes were produced and supplied by Respire Diagnostics Ltd. Microfluidic diagnostic hydrophilic film, 9901PB and microfluidic diagnostic tape 9972A, was obtained from 3 M and laser-cut to shape in house. A custom-made tool for aligning the capillary fill device was manufactured and provided by Respire Diagnostics Ltd. Corning 96-well clear polypropylene plates were supplied by Fisher Scientific.

### Instrumentation

A PalmSens4 potentiostat was used for all electrochemical measurements. A Fluostar Omega multimode microplate reader was used for absorbance measurements. The microfluidic diagnostic tape and hydrophilic film were cut using a Universal Laser System VLS2.30DT. A VWR precision balance (model SWC13.01, *d* = 0.01 mg) was used for all of the weight measurements.

### Electrode Preparation

Thionine and methylene blue sensors were prepared as followed: A 50 mM HEPES buffer was adjusted to pH 7.8 using 0.1 M sodium hydroxide and 0.1 M hydrochloric acid. Trehalose (30 mg/mL), hypoxanthine (100 mM), and either thionine or methylene blue were then added to the buffer to a final concentration of 10 mM. The mixture was heated at 60 °C for 1 h, followed by 1 h of ultrasonication in an ultrasonic bath. Subsequently, 10 μL of the resulting solution was drop-cast onto the working electrode of a disposable glassy carbon electrode and dried by heating at 60 °C for 1 h. For point-of-care analysis, the modified sensor was incorporated into a capillary-fill device. After modifying the disposable carbon electrode, double sided thick spacer tape (9972A by 3M) and diagnostic hydrophilic film (9901PB by 3M) were aligned using the aligning tool custom-made by Respire Diagnostics.

### Urine Sample Analysis and Ethical Approval

To assess the sensor in a real urine matrix, 25 patient urine samples submitted to the diagnostic laboratory for routine testing and surplus to diagnostic requirement, were spiked with diagnostically relevant concentrations of xanthine oxidase (between 0 and 10,000 U/L). The samples were tested immediately after spiking to minimize XO degradation, using both a commercially available colorimetric XO assay and the developed XO sensor. The samples were diluted accordingly to fall within the manufacturer’s dynamic detection range, and were used undiluted for quantification with the developed XO sensor. Ethical approval for this study was granted by the West London Research Ethics Committee (IRAS 162013, 06/Q0406/20). The protocol allows the use of clinical samples routinely collected for diagnostic purposes, even in cases where written patient consent is not typically obtained.

### Electrochemical Measurements

Artificial urine (AU) was prepared according to Sarigul et al.^21^ to mimic human urine composition. The electrochemical behavior of thionine and hypoxanthine on a 3 mm diameter glassy carbon rod electrode was assessed in nitrogen-purged AU. CV curves at a range of scan rates (3, 6, 10, 30, 100, 600, and 1000 mV/s) were recorded with and without XO.

For electrochemical quantification of XO, a chronoamperometric measurement was performed in two steps: first, an equilibration step at a potential less positive than the expected oxidation potential of leucothionine (−0.15 V), and second, a measurement step at a potential higher than its oxidation potential (+0.15 V). The second step allows for the detection of XO activity through the oxidation of leucothionine. The recorded current was integrated over time to reduce random noise, and the resulting charge plotted against edge-corrected time to account for edge effects, as described by Hildebrand et al.^23^ The final charge for each concentration and time interval was calculated as the average of the last 10 s of the measurement. The incubation time of the XO-containing sample with the sensor was optimized by recording chronoamperometric signals after defined time intervals (0, 3, 10, 15, and 20 min after filling) and choosing signal and background combination resulting in a calibration with highest *R* ^2^. XO containing solutions were prepared fresh from stock for each calibration. Limit of detection (LOD) was calculated as 3σ/slope for each calibration.

### Capillary Fill Device

The capillary fill device consisted of a spacer layer and a hydrophilic lid. The thickness and geometry of the spacer layer determined the total fill volume of the device. The capillary fill devices were assembled using an alignment tool (Figure 2a) by first aligning the precut double-sided spacer tape with the inlet, reservoir, and outlet onto the modified disposable glassy carbon electrode (Figure 2b, bottom). Then, a hydrophilic layer with an air outlet was attached on top of the spacer layer (Figure 2b, top). For measuring, the assembled device is dipped into a urine sample until the chamber is filled (3 s, Figure 2c). For electrochemical measurements, the sensor is connected to pins of a PalmSens4 potentiostat (Figure 2d) or a hand-held potentiostat (Figure 2e). Filling repeatability and accuracy were evaluated by comparing the theoretical fill weight, calculated as the fill area multiplied by the spacer thickness, with the actual measured weight after filling the device with deionized water. For point-of-care sample analysis, a hand-held potentiostat, such as a PalmSens Sensit Smart, can be used.

**Figure 2:**
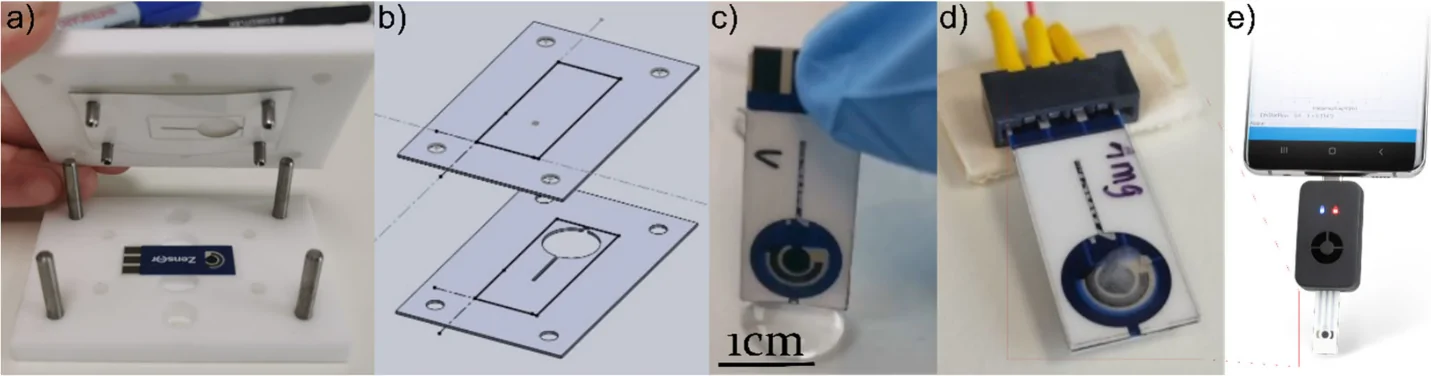
(a) Alignment of spacer layer and hydrophilic lid using an alignment tool. (b) Visual representation of spacer layer with short inlet, reservoir, and air outlet (bottom) and hydrophilic lid with air outlet (top). (c) The device is dipped into a sample until the chamber is filled. (d) For electrochemical measurements, the disposable glassy carbon sensor is connected to pins of a potentiostat (e) or a hand-held potentiostat. Photograph in (e) courtesy of PalmSens, Copyright 2025.

## Results and Discussion

### Electrochemical Behavior of Thionine Acetate with XO and Hypoxanthine in AU

According to Henry’s law, the concentration of oxygen in the capillary fill device is 0.259 mmol*dm^–3^ (at 298 K and 101,325 Pa). One unit of XO is defined as the amount of enzyme catalyzing one μM of substrate per minute.^24^ Considering the proposed reaction in Figure 1, 10,000 U/L XO consume 10 mM hypoxanthine and therefore 10 mM of oxygen per minute. At the highest measured XO concentration, the dissolved oxygen was calculated to be consumed within 0.78 s. The surface area in contact with ambient air in the capillary fill device is the outlet (0.5 mm × 0.5 mm) and the inlet (1 mm × 0.21 mm) totalling in 0.46 mm^2^ area, which is too small for oxygen to be replenished at the rate which it is consumed.

The oxygen-limited regime in the capillary fill device was confirmed through XO quantification on PEDOT:PSS electrodes. When XO reacts in an open sensor format, enzymatic H_2_O_2_ generation can be readily quantified on PEDOT:PSS electrodes; however, this signal is absent inside the capillary fill device (Supporting Information, Figure 2), consistent with the rapid depletion of dissolved oxygen. Controlled additions of exogenous H_2_O_2_ confirmed that electrochemical detection itself remains functional within the capillary fill device (Supporting Information, Figure 3), which confirms the replenishment of oxygen as the limiting factor.

To verify that the resulting electrochemical response relies strictly on catalytic substrate turnover rather than background shifts or nonspecific matrix reactions, key negative controls were performed (Supporting Information, Figure 4) in the open sensor format on PEDOT:PSS electrodes. In the presence of 10,000 U/L XO and 100 mM hypoxanthine, chronoamperometric measurements recorded at −0.25 V exhibit a characteristic cathodic current response. Conversely, controls lacking either substrate (0 mM hypoxanthine) or enzyme (0 U/L XO) show no current response and rapidly decay to baseline, confirming that signal generation depends exclusively on active enzymatic turnover.

Under the oxygen-free conditions as expected in the capillary fill device, XO reduces thiAc to Lthio, which can be oxidized and quantified electrochemically. When comparing thiAc to methylene blue under oxygen-free conditions, thiAc showed a more distinct relative increase in oxidation current over its baseline (Supporting Information, Figure 5). To investigate the electrochemical reaction, CVs were recorded at scan rates ranging from 3 to 1000 mV/s on 3 mm diameter glassy carbon rod electrodes with 100 mM hypoxanthine and 10 mM thiAc in nitrogen-purged AU (Figure 3a). Oxidation peaks at different scan rates plotted against the logarithm of the scan rate reveals a linear correlation with a slope of 0.556 and *R* ^2^ of 0.994 (Figure 3a, inlet), indicating that thiAc is diffusing freely to the electrode surface according to Randles Sevčik equation.^25^

**Figure 3:**
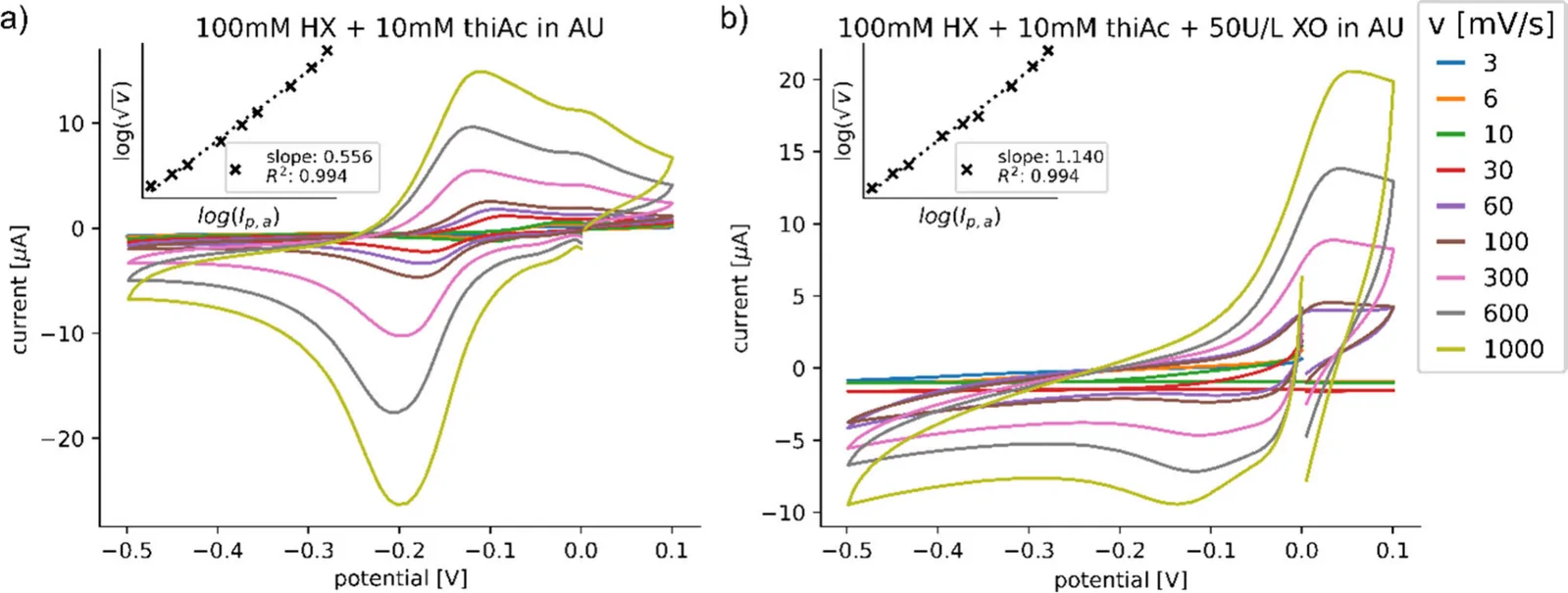
(a) CVs were recorded for scan rates ranging from 3 to 1000 mV/s on a glassy carbon rod electrode in nitrogen purged AU with 100 mM hypoxanthine and 10 mM thionine dissolved. Oxidation peak vs log of scan rate reveals a linear correlation with slope 0.556, indicating thiAc is diffusing freely to the surface. (b) Addition of an intermediate concentration of XO, 50U/L, results in an increased oxidation peak and decreased reduction peak, as XO uses thiAc as secondary electron acceptor. HX, hypoxanthine; thiAc, thionine acetate; AU. artificial urine; XO, xanthine oxidase.

The addition of an intermediate concentration of XO (50 U/L) results in an increased oxidation peak and a decreased reduction peak, as XO utilizes thiAc as a secondary electron acceptor (Figure 3b). XO present in urine, when supplied with an excess of its substrate hypoxanthine, therefore leads to the reduction of thiAc in solution. This electrochemical signal can be used for the quantitative detection of XO activity.

### Electrochemical Quantification of XO in Artificial Urine

Signal acquisition for XO quantification is shown in Figure 4. The incubation time of the XO-containing sample with the sensor was optimized by recording chronoamperometric signals after defined time intervals and choosing signal and background combinations resulting in a calibration with the highest *R* ^2^. XO containing solutions were prepared fresh from stock for each calibration. The final charge for each concentration and time interval was calculated as the average of the last 10 s of the measurement.

**Figure 4:**
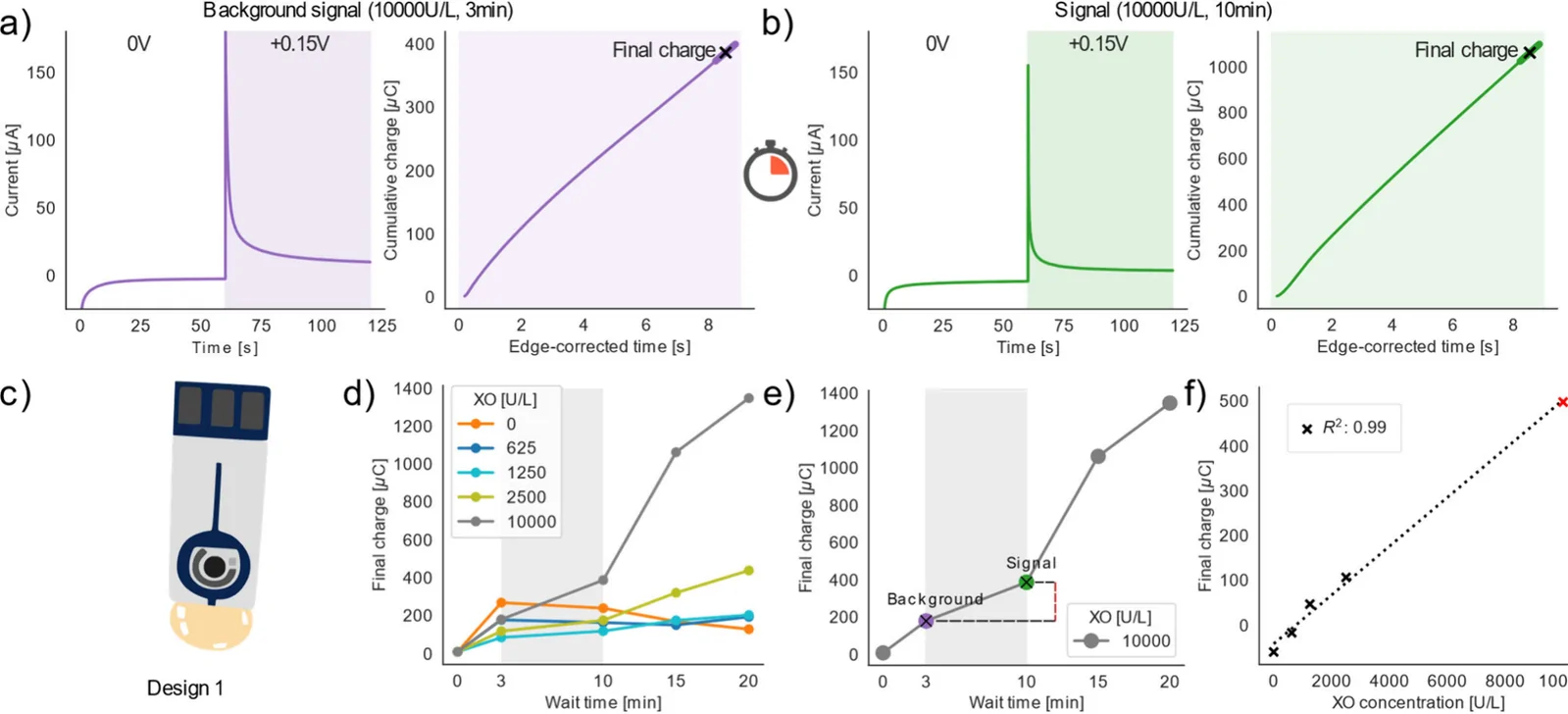
Electrochemical XO detection. (a) A chronoamperometric measurement step which at +0.15 V is taken directly after capillary fill sensor is filled and (b) after different incubation times. The recorded current at +0.15 V was integrated over time to reduce noise, and the mean of the final 10 s of signal were used as final signal. (c) The signal acquired using design 1 is shown in this figure, the sensor design was later optimized for shorter time-to-result and smaller intersensor variability. (d) The final charge was measured for 5 concentrations at different incubation steps. (e) The optimal background and signal combination resulting in the smallest *R* ^2^ was found to be 3 and 10 min (shaded gray area). (f) The resulting background-corrected final charges result in a linear calibration with *R* ^2^ of 0.998.

For a proof of concept, the final charge after 0, 3, 10, 15, and 20 min detected by 5 single-use capillary fill sensors are shown in Figure 4d. The optimal background-to-signal combination with the highest *R* ^2^ of 0.9985 and a limit of detection (LOD) of 343.8 U/L was found to be a background signal recorded at 3 min and a signal recorded at 10 min (Figure 4f). The resulting background-corrected charges and calibration curve are shown in (Figure 4f). The time to result was minimized by design optimization for faster and more adequate filling.

### Design and Signal Acquisition Optimization

It was observed that air bubble formation in the capillary fill device led to incomplete substrate dissolution and inconsistent results. Additionally, 10 min wait time for signal acquisition was too long. Subsequently, the sensor design was optimized for repeatability, accuracy, and signal acquisition time, with the aim of minimizing the influence of inadequate filling on the sensor response. Figure 5a) shows initial design 1 with subsequently developed designs 2 and 3, optimized for enhanced filling reliability. The spacer layer and hydrophilic lid are shown separately and assembled into the final device. A photograph of an assembled sensor is shown alongside each design. To assess the repeatability and accuracy of capillary fill performance, the mean fill factor (μ), defined as the ratio of measured to expected weight, and the coefficient of variation (CV) of fill weight across five devices were determined (Figure 5b). A μ < 1 indicates incomplete filling, as expected due to a small volume of dried substrate remaining on the working electrode. In design 1, minimal air exhaust led to trapped air, a lower mean fill volume (μ = 0.95), and high fill variability (CV = 12.8%). Design 2, featuring very large air exhaust, exhibited a higher mean fill factor (μ = 0.98); however, liquid overflow (μ > 1) occurred in 2 devices, leading to moderately high variability (CV = 7.4%). Design 3 showed the most consistent performance, with a mean fill factor of 0.96 and low variability (CV = 2.1%) and was therefore selected as the final design. The resulting calibration curves for low concentrations of XO (0–250 U/L) are shown in Figure 5c. Design 3 shows the highest sensitivity of 1.215 μC*L/U and *R* ^2^ of 0.87, and a LOD of 3.62 U/L.

**Figure 5:**
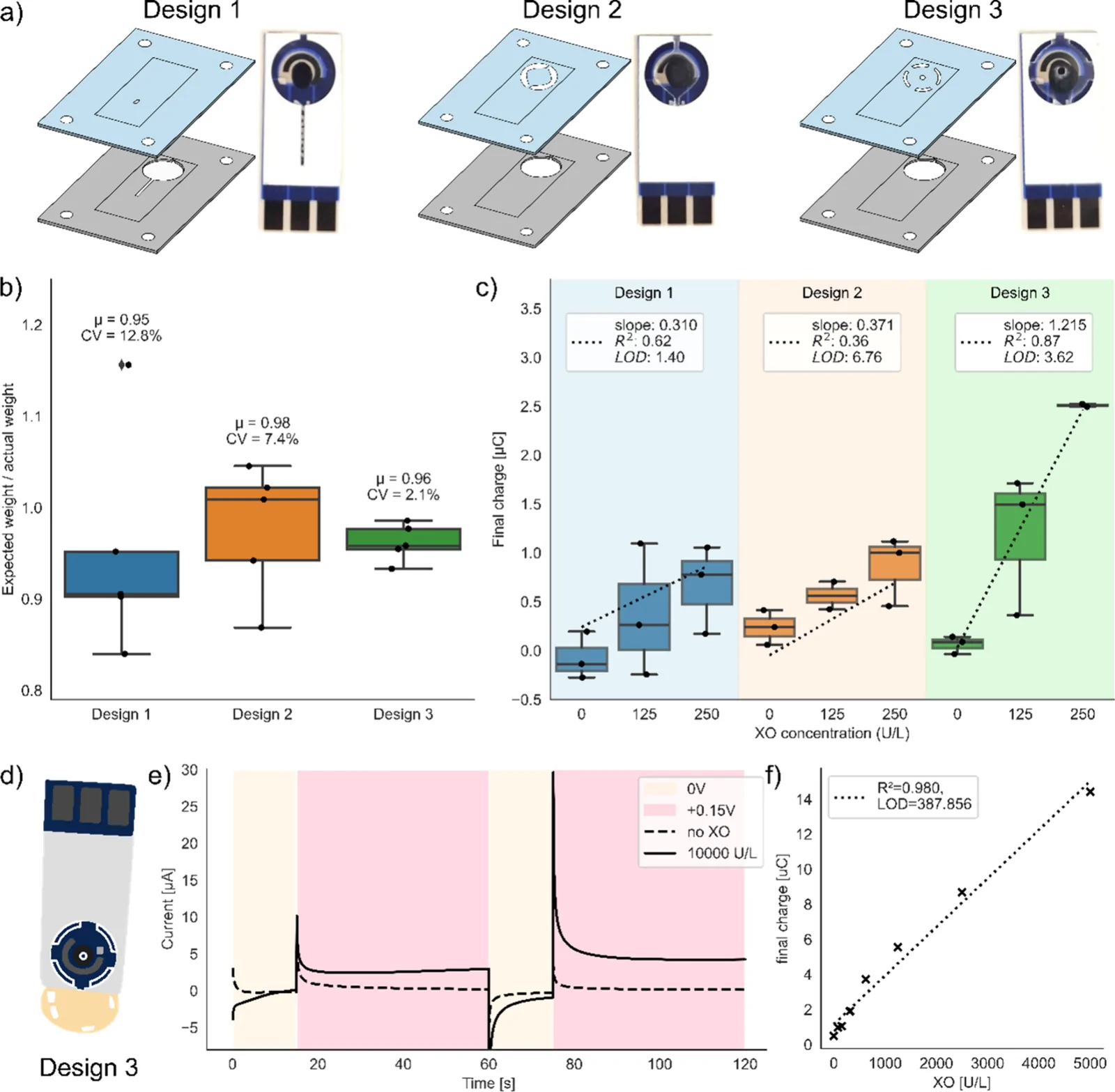
(a) Three different capillary fill device designs were tested for optimal filling and sensing performance. The spacer layer for each design is shown in gray, and the hydrophilic lid with air exhaust in light blue. The assembled sensor is shown alongside. Design 1 has one small air exhaust at the end of an exhaust channel, design 2 air exhausts around the working electrode, and design 3 an additional small air exhaust smaller than and directly above the working electrode. (b) The capillary fill devices were weighed before and after filling to assess fill accuracy and repeatability. The ratio of the expected fill weight (calculated as fill area multiplied by spacer thickness) to the actual measured weight, μ, was below 1 for all designs (μ = 0.95 for design 1, μ = 0.98 for design 2, μ = 0.96 for design 3). Design 3 exhibited the lowest coefficient of variation of fill weight (2.1%) compared to design 1 (12.8%) and design 2 (7.4%). (c) Three concentrations of XO were tested using nine sensors per design. Design 3 exhibited the highest *R* ^2^ (0.87) and slope (1.215 μC*L/U), with a limit of detection (LOD) of 3.62 U/L. (d) Each measurement was started immediately after dipping the device into urine. (e) Equilibrium and measurement steps were applied sequentally for faciliate testing. (f) The measurement sequence resulted in a linear calibration with *R* ^2^ of 0.98 for design 3.

To facilitate testing, the equilibrium and measurement steps for design 3 (Figure 3d) were applied sequentially as shown in Figure 5e. The steps were shortened due to faster filling and signal development in design 3 (15 s equilibrium at 0 V, 60 s signal at +0.15 V). As previously, background and signal combinations were compared and found to be cycle 0 for background (60 s) and cycle 1 for signal (120 s) (Figure 5e). The background-corrected final charge of 8 individual sensors resulted in a linear calibration in AU with an *R* ^2^ of 0.980 (Figure 5f).

### Matrix Effects

To evaluate potential matrix effects in real samples, the impact of key urinary interferents, uric acid, creatinine, urea, ascorbic acid, and nitrofurantoin (a commonly used antibiotic for uncomplicated UTIs), was assessed across physiological concentration ranges using cycling voltammetry (Supporting Information, Figure 6). For each interferent, the percentage change in peak potential (***E***_**p**_) and cathodic peak height (***I***_**p**_) was compared directly to a blank PBS solution (Supporting Information, Figure 7). The percentage difference was calculates as

$$\mathrm{percent change}=\frac{\left(\mathrm{sample value}-\mathrm{PBS value}\right)}{\mathrm{PBS value}}\times 100\%$$

Peak potential shifts remained low (<4.9%) across most conditions, although higher changes in peak current were observed at upper physiological concentration limits for urea (50–500 mM; 28.6% change) and ascorbic acid (0.1–0.8 mM; 36.8% change), while solution pH remained stable (7.0 ± 0.34) (Supporting Information, Figure 3). Similarly, evaluating the sensor at varying pH levels (pH 3.0, 6.0, and 8.0, Supporting Information, Figure 8) revealed minimal peak potential shifts within the physiological window (+9.5% at pH 6.0 and +0.0% at pH 8.0 relative to baseline PBS), while maintaining robust electroactive responses with peak current increases of +45.1% and +51.3%, respectively (Supporting Information, Figure 9). Because the sensor relies on a differential signal correction mechanism (***S***_**120**s_ – ***S***_**60**s_), background matrix variations are subtracted out, helping to account for these peak height differences during enzyme quantification.

### Sensor Validation in Urine Samples

Figure 6 demonstrates the XO activity measured using the XO sensor and a commercially available XO assay in spiked human urine samples. The XO assay is based on the oxidation of hypoxanthine to superoxide by XO. Superoxide subsequently forms a formazan die when reacting with tetrazolium salt (WST-8), which is quantified colorimetrically at 450 nm.^22^ A statistically significant linear correlation was observed between XO concentration (calculated from the spiked XO weight) and absorbance measured at 450 nm (*R* ^2^ = 0.911, *p* < 0.001; Figure 6a), as well as between XO concentration and the final charge measured by the developed sensor (*R* ^2^ = 0.798, *p* < 0.0001, Figure 6b) in 25 spiked human urine samples. To evaluate method agreement, a Bland–Altman analysis was conducted (Supporting Information, Figure 10), revealing a mean bias of 893.86 U/L with 95% limits of agreement ranging from −5304.20 to +3516.47 U/L. Furthermore, spike-and-recovery analysis across four concentration intervals demonstrated acceptable mean analytical recoveries ranging between 90.3% and 101.9% across all evaluated ranges: Low (1074–3007 U/L; μ = 90.3%, %RSD = 36.7%), Mid-Low (3007–5753 U/L; μ = 93.4%, %RSD = 58.9%), Mid-High (5753–9082 U/L; μ = 101.9%, %RSD = 18.4%), and High (9082–13555 U/L; μ = 92.4%, %RSD = 35.0%) (Supporting Information, Figure 11). Given that urine containing *E. coli*, the primary uropathogenic in UTIs, exhibits a mean XO activity of >10,000 U/L (Supporting Information, Figure 1), the sensor demonstrates sufficient precision to differentiate low baseline levels in uninfected urine from the elevated XO activity characteristic of infection.

**Figure 6:**
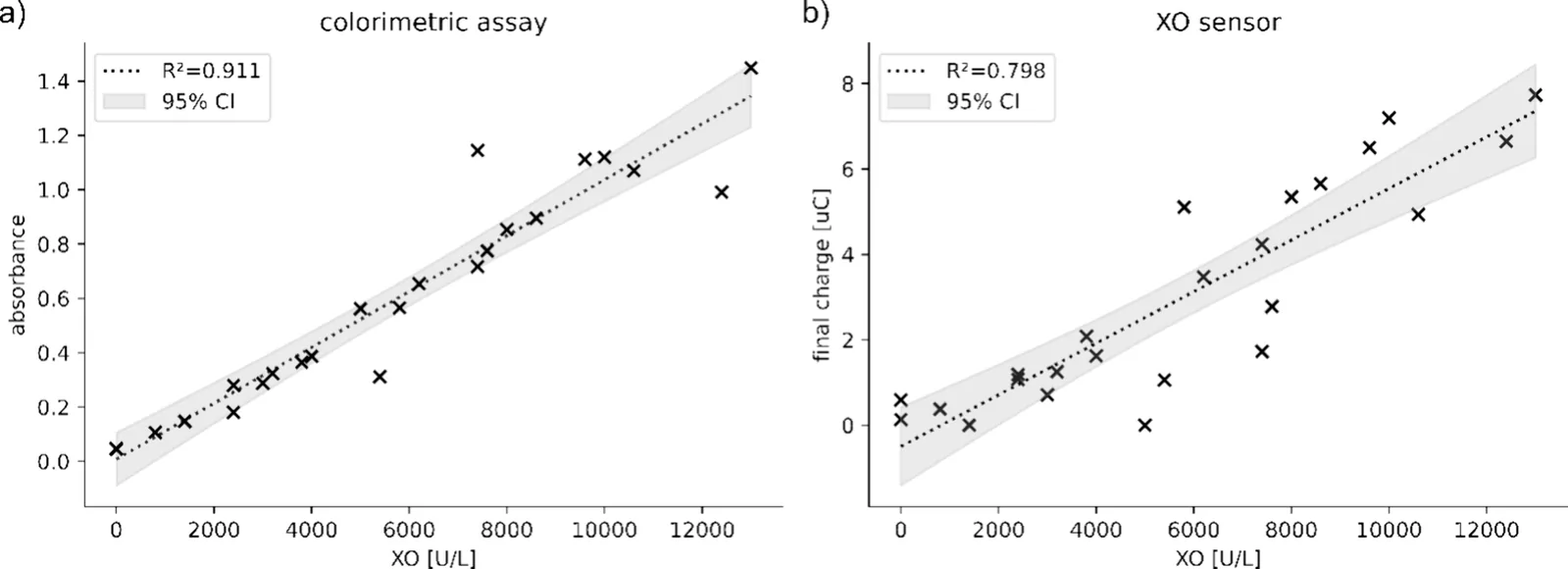
Twenty-five patient urine samples from different patients were spiked with clinically relevant XO concentrations and tested immediately with commercially available colorimetric XO assay and capillary fill sensors. (a) Correlation between spiked XO concentration and absorbance using a commercial colorimetric XO assay (*R* ^2^ = 0.911, *p* < 0.0001). (b) Correlation between spiked XO concentration and the final charge measured by the developed XO sensor (*R* ^2^ = 0.798, *p* < 0.0001). Both assays show statistically significant linear correlations, demonstrating the sensor’s performance in clinical urine samples.

## Conclusion

In this study, we developed a sensing principle to measure the activity of the enzyme XO in a point-of-care capillary fill sensor. For this, the substrate hypoxanthine, secondary electron acceptor thionine acetate, and trehalose for facilitated dissolution were dried onto the working electrode of disposable glassy carbon electrodes. In their dried state, the used chemicals are not expected to degrade, which supports sensor stability. Signal acquisition involved recording a sequence of equilibration and oxidation steps to obtain a background and a measurement signal. The proposed oxidation potential and electrochemical behavior of thionine acetate on the disposable glassy carbon electrode was assessed using cyclic voltammetry, indicating a simple diffusion-controlled process. The background to measurement signal combination was found by comparing different time-combinations, and the capillary fill device was optimized for fill accuracy and repeatability, resulting in a 2.1% variation in fill weight, *R* ^2^ of 0.985 across the relevant XO concentration range and LOD of 343.8 U/L. Finally, testing in spiked human urine samples demonstrated mean recoveries ranging between 90.3% and 101.9% across evaluated concentration intervals (1074–13,555 U/L), yielding a linear correlation between final charge and XO concentration with an *R* ^2^ of 0.798. The elevated variance observed in the Mid-Low concentration group (%RSD = 58.9%) was attributed to individual sample outliers rather than an intrinsic signal-to-noise limitation of the sensor, as the lowest concentration group exhibited lower relative variation (%RSD = 36.7%). Altogether, these findings demonstrate the performance of the sensor in human urine matrices and establish a solid foundation for point-of-care XO diagnostics.

While longitudinal stability studies were not conducted in this preliminary work, the novelty of this sensing principle is defined by its dry reagent format, enabling enzyme activity quantification in the low-oxygen environment in the capillary fill device. While long-term stability and clinical patient validation were beyond the immediate scope of this work, these findings demonstrate clear proof of concept for point-of-care XO detection in human urine.

We demonstrated the calibration-free, sensitive detection of XO in the clinically relevant concentration range using a simple and easy-to-use capillary fill device. By choosing suitable substrates and secondary electron acceptors, this sensing principle could be applied to other enzymes in the future.

## Supplementary Material



## Notes

Reported XO concentrations in sterile and infected urine across studies; comparison of open-system O_2_-dependent sensing versus thionine-mediated sensing inside the capillary fill device; cyclic voltammetry of direct H_2_O_2_ additions on open PEDOT:PSS electrodes vs inside the capillary fill device; chronoamperometric negative controls evaluating sensor response in the presence and absence of hypoxanthine and xanthine oxidase; side-by-side electrochemical comparison of methylene blue and thionine acetate as redox mediators for xanthine oxidase; electrochemical evaluation of common urinary interferences via cyclic voltammetry; quantitative percent difference comparison of peak current and potential across urinary interferents; cyclic voltammograms evaluating sensor response across varying pH conditions (pH 19 3, 6, and 8); quantitative analysis of peak potential and peak current variations across pH ; and Bland–Altman agreement analysis comparing the capillary-fill electrochemical sensor against commercial colorimetric assay; spike and recovery analysis of human urine samples (PDF)

## ¶

R.A.: Weill Cornell Medicine, 8C9R+735, Education City, Al Luqta St, Al-Rayyan, Katar
