# Supplementary material for: Rapid Point-of-Care Quantification of Urinary Tract Infection Biomarker Xanthine Oxidase
Source: ACS Omega. 2026 Oct 28;11(44):69381–8. doi: 10.1021/acsomega.6c10153 (PMC13757073; doi:10.1021/acsomega.6c10153)
Supplement: Supplementary file 1 [file ao6c10153_si_001.pdf]

Rapid point-of-care quantification of urinary tract infection biomarker xanthine oxidase – Supplementary Information

Ellen Stadler[1,2,3], Mark Sutton[7,8], Colin Brown[9], Wubbo de Boer[6], †Rahaf Ammouri[6], Alison Holmes[1,2,3,4,5], Timothy M Rawson[1,2,3,4,5], Danny O`Hare[1,6]

1: Centre for Antimicrobial Optimisation, Imperial College London, W12 0NN London, UK. **2:** Department of Infectious Diseases, Imperial College London, SW7 2AZ London, UK. **3:** National Institute for Health Research, Health Protection Research Unit in Healthcare Associated Infections and Antimicrobial Resistance, Imperial College London, SW7 2AZ London, UK. **4:** David Price Evans Infectious Diseases & Global Health Group, The University of Liverpool, L69 7BE Liverpool, United Kingdom. **5:** Fleming Institute, Imperial College London, SW7 2AZ London, UK **6:** Department of Bioengineering, Imperial College London, London SW7 2AZ, United Kingdom. **7:** Antimicrobial Discovery, Development and Diagnostics (AD3) UK Health Security Agency, Porton Down, SP4 0JG Salisbury, United Kingdom. **8:** Institute of Pharmaceutical Science, King's College London, WC2R 2LS London, United Kingdom. **9:** Healthcare Associated Infections, Fungal, Antimicrobial Resistance, Antimicrobial Use, and Sepsis Division, UK Health Security Agency, E14 4PU London, United Kingdom.

KEYWORDS: Clinical diagnostics, urinalysis, enzyme biosensor, xanthine oxidase, electrochemistry, point-of-care sensor

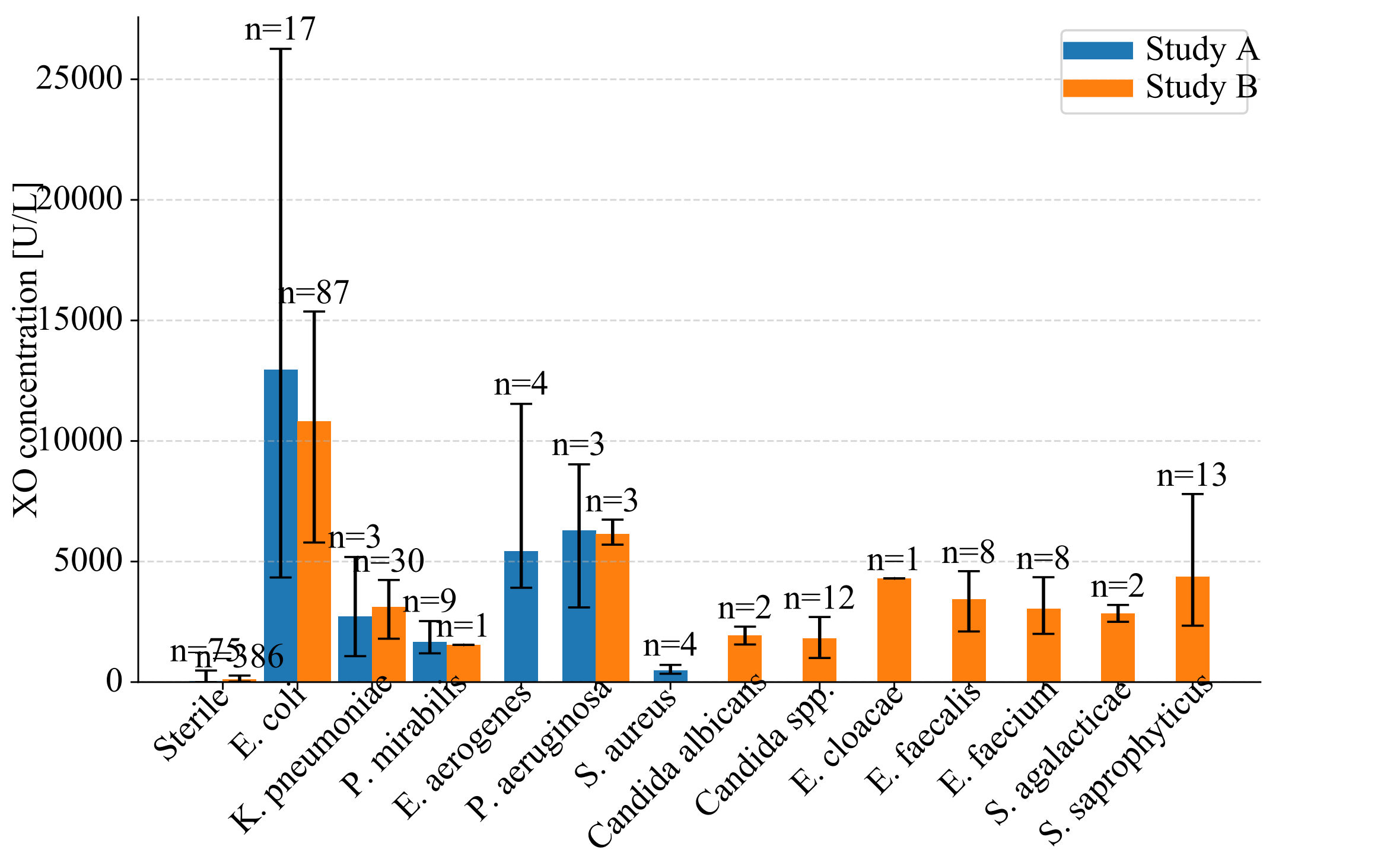

S 1: Reported XO concentrations in sterile and infected urine from two studies looking at XO as a biomarker for UTIs. Height of bars indicating mean, error bars minimum and maximum reported XO concentrations. For sterile samples and samples with reported E. coli, K. pneumoniae, P. mirabilis, data from both study A [1] and study B [2] are available. Statistical analysis was not conducted due to lack of raw data.

† Weill Cornell Medicine, 8C9R+735، Education City, Al Luqta St, Al-Rayyan, Katar

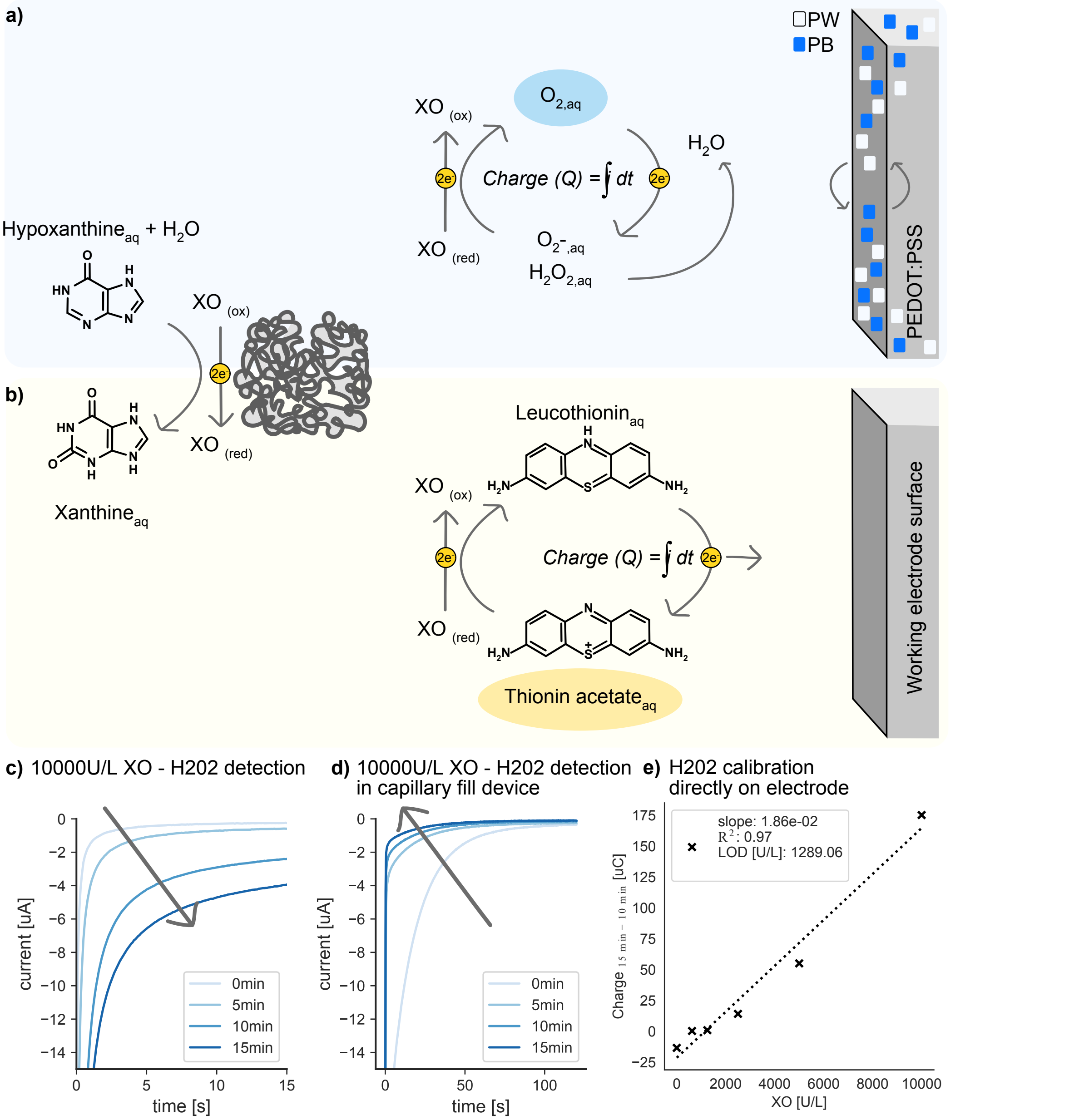

S2: Comparison of open-system O_2-dependent sensing versus thionin-mediated sensing in the capillary fill device. a) Schematic representation of the O_2-dependent enzymatic reduction pathway in an open sensor system, where XO uses ambient dissolved oxygen to generate H_2O_2, which is catalytically reduced at the PEDOT:PSS working electrode surface. b) Schematic of the oxygen-limited sensing mechanism inside the capillary fill device, where thionine acetate acts as the dominant secondary electron acceptor. c) Chronoamperometric traces at -0.25 V following the addition of XO $(10{,}000\ U/L)$ to an open-system PEDOT:PSS electrode containing pre-dried hypoxanthine, showing increasing cathodic currents due to H_2O_2 production. d) Chronoamperometric traces at -0.25 V for the same reaction inside the enclosed capillary fill device, where currents quickly return to baseline, pointing toward rapid oxygen depletion in the closed volume. e) Calibration plot of charge difference $(Q_{15\ min} - Q_{10\ min})$ versus XO activity ($0 - 10{,}000\ U/L$) in the open sensor format ($R^2 = 0.97$), demonstrating reliable quantification of XO activity via H_2O_2 reduction in the presence of continuous O_2.

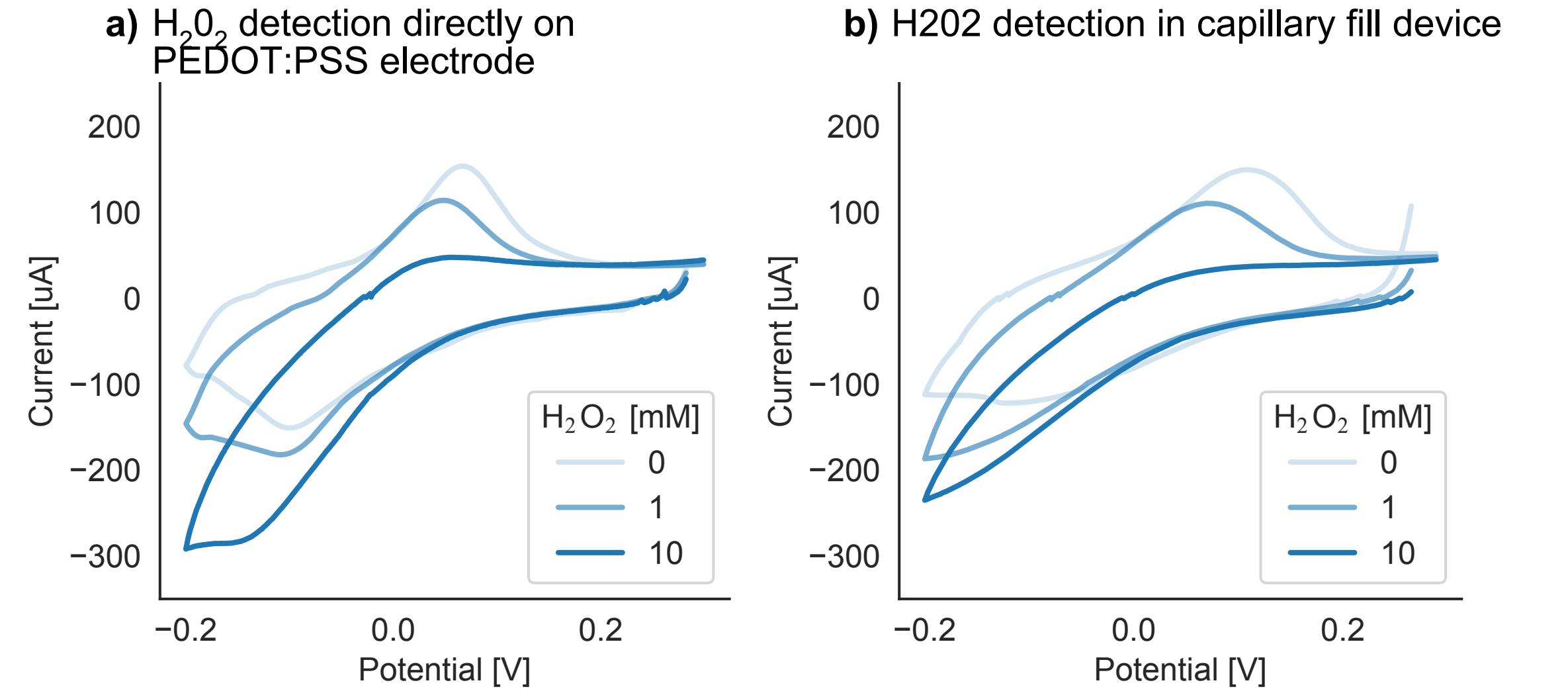

S3: Cyclic voltammetry of direct H_2O_2 additions on PEDOT:PSS. a) Cyclic voltammograms (scan rate 100mV/s) recorded directly on an open PEDOT:PSS electrode with increasing concentrations of H_2O_2 $(0, 1,$ and $10 mM)$. b) Cyclic voltammograms recorded under identical conditions inside the enclosed capillary fill device, demonstrating that H_2O_2 redox activity remains detectable within the capillary architecture.

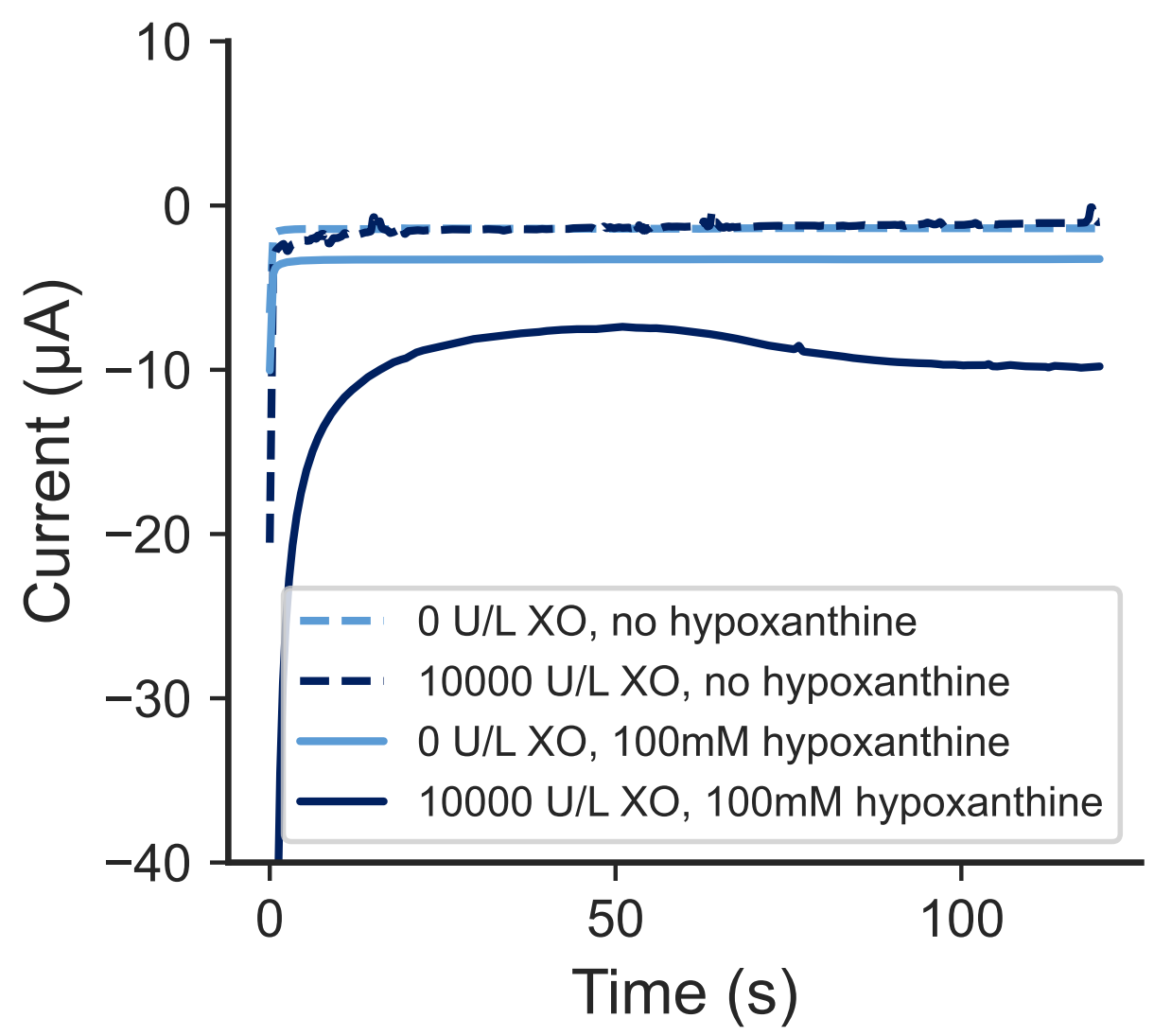

S4: Chronoamperometric measurements in the open sensor format, reducing H_2O_2 on PEDOT:PSS sensors, recorded at -0.25 V comparing sensors pre-modified with 100 mM hypoxanthine against unmodified sensors in the presence ($10{,}000$ U/L) and absence (0 U/L) of xanthine oxidase (XO). Upon enzyme addition, a pronounced increase in cathodic current is observed exclusively when substrate (hypoxanthine) is present ($10{,}000$ U/L XO, 100 mM hypoxanthine), confirming that signal generation depends strictly on enzyme-substrate turnover rather than non-specific baseline shifts.

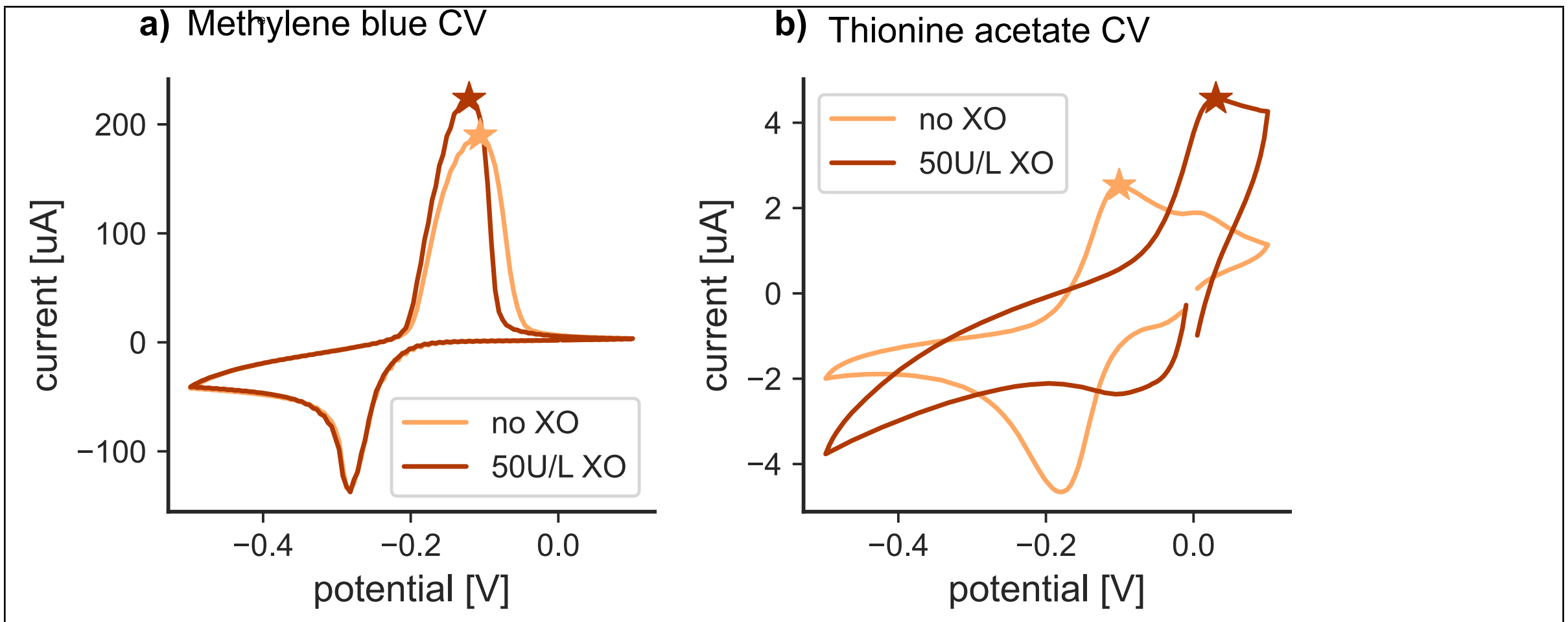

S5: Electrochemical comparison of redox mediators for xanthine oxidase (XO). a) Cyclic voltammograms (100mV/s) of 100mM methylene blue in N_2-bubbled artificial urine containing 10mM hypoxanthine, with (50U/L) and without XO, showing a high background current with a modest relative increase upon XO addition. b). Cyclic voltammograms recorded under identical N_2-bubbled conditions for 100mM thionine acetate. After adding XO, a clear catalytic signal is observed, shown by a smaller reduction peak and a near doubled oxidation peak (marked by stars). Although the absolute current magnitude for thionine acetate is lower, the significant change in peak current (from 2 to 4 μA) confirms its good performance as an electron acceptor without oxygen.

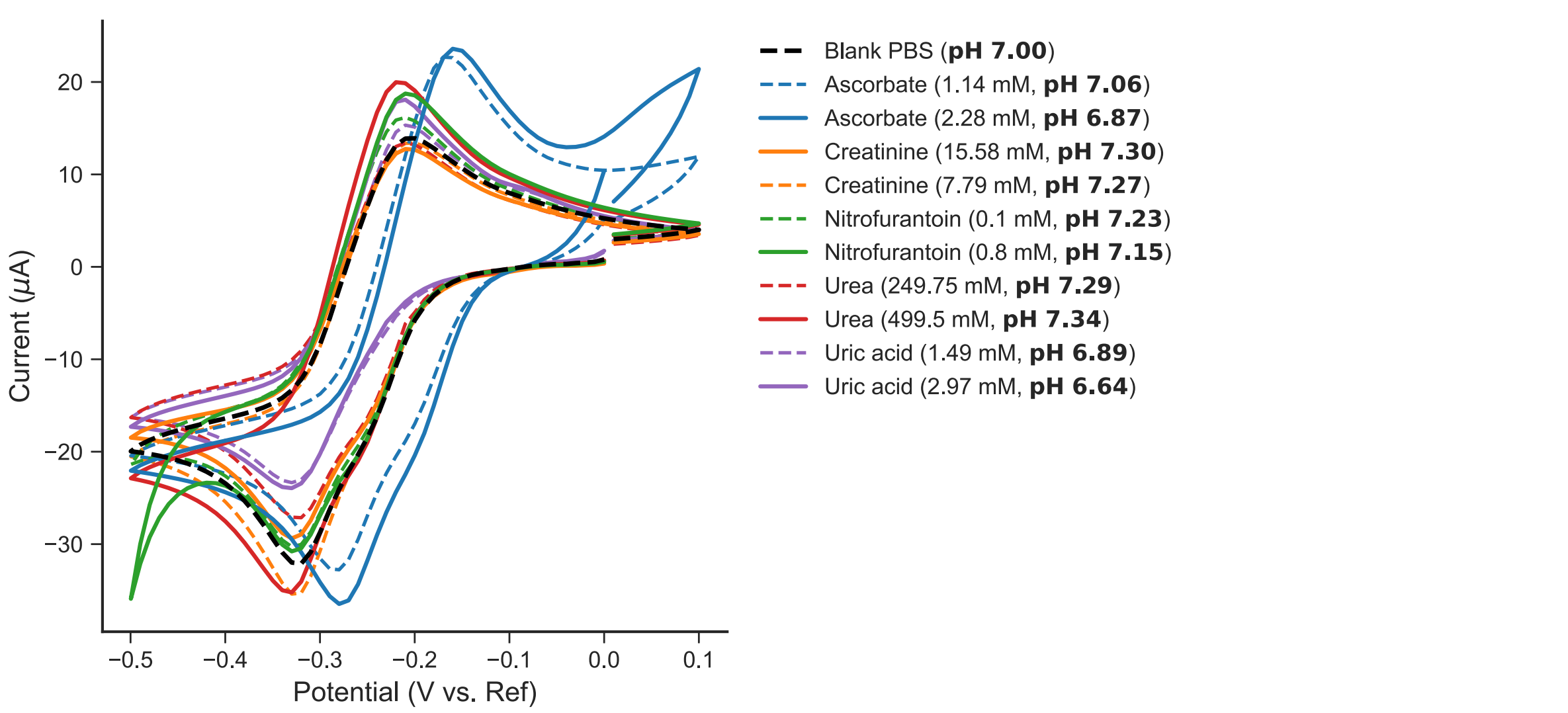

S6: Effect of urinary interferents on the redox reaction of thionine acetate to leucothionine. Cyclic voltammograms (CVs) recorded in 100mM thionine acetate dissolved in 0.1M PBS (pH 7, black dashed line) and in the presence of common urinary interferents spiked at physiological concentrations: Ascorbate (1.14mM and 2.28mM), Creatinine (7.79mM and 15.58mM), Nitrofurantoin (0.1mM and 0,8mM), Urea (249.75mM and 499.5mM), and Uric acid (1.49mM and 2.97mM). Post-addition solution pH values are indicated in parentheses.

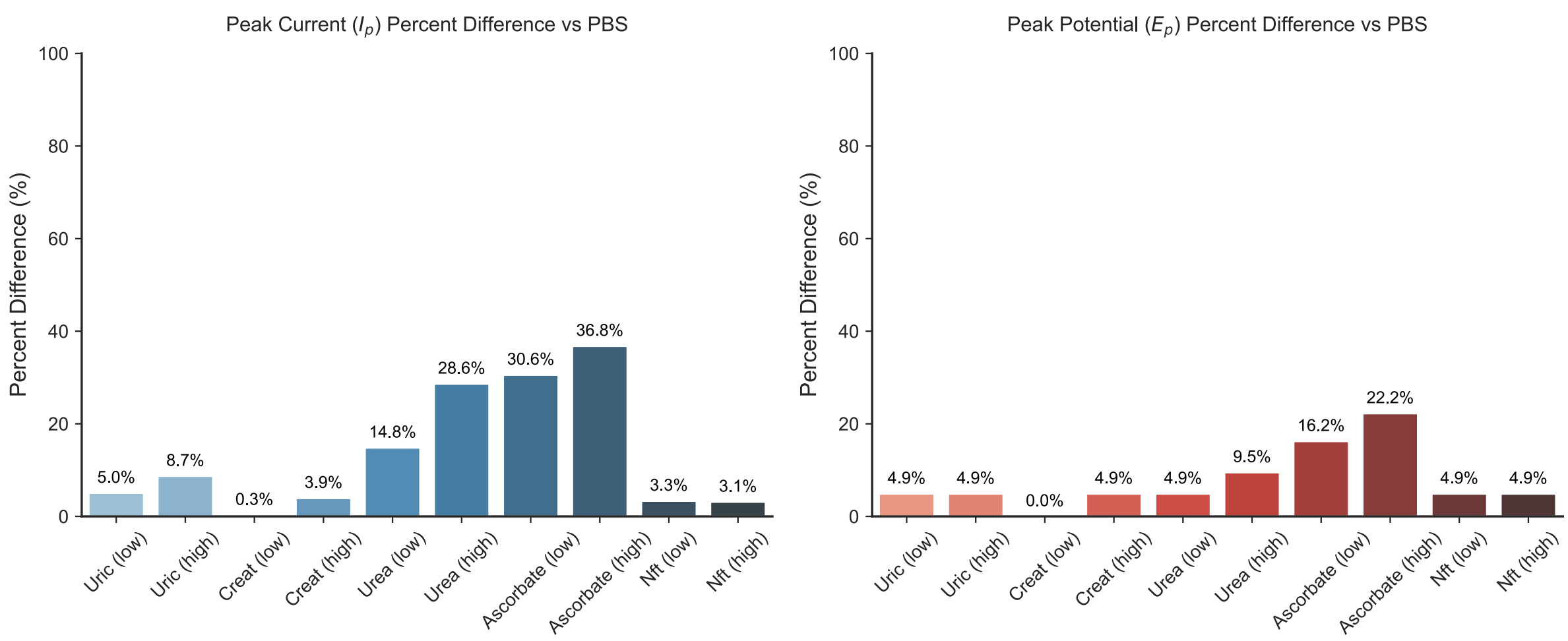

S7: Quantitative comparison of peak current and potential shifts across urinary interferents. Percentage differences relative to blank PBS baseline for (left) cathodic peak current (I_p) and (right) peak potential (E_p) following the addition of low and high physiological concentrations of uric acid, creatinine (Creat), urea, ascorbate, and nitrofurantoin (Nft).

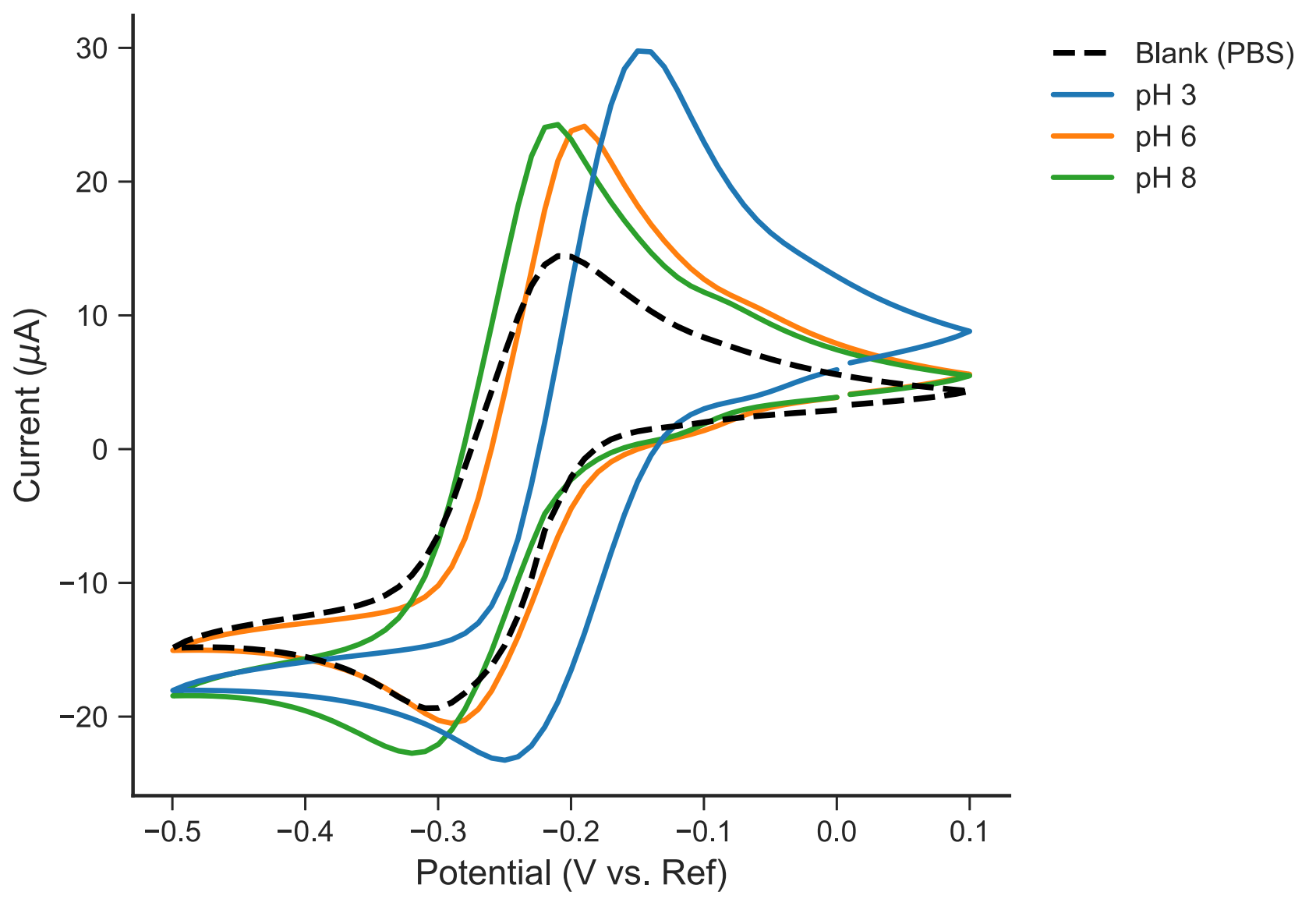

S8: Cyclic voltammograms (CVs) recorded on carbon electrodes modified with thionine acetate, hypoxanthine, and trehalose in blank PBS (dashed black line) and in PBS solutions adjusted to pH 3.0 (blue), pH 6.0 (orange), and pH 8.0 (green). Measurements were taken over a potential range of -0.5 V to +0.1 V (vs. Ref) to evaluate peak potential (E_p) shifts and peak current (I_p) behavior of the thionine redox couple across varying pH conditions.

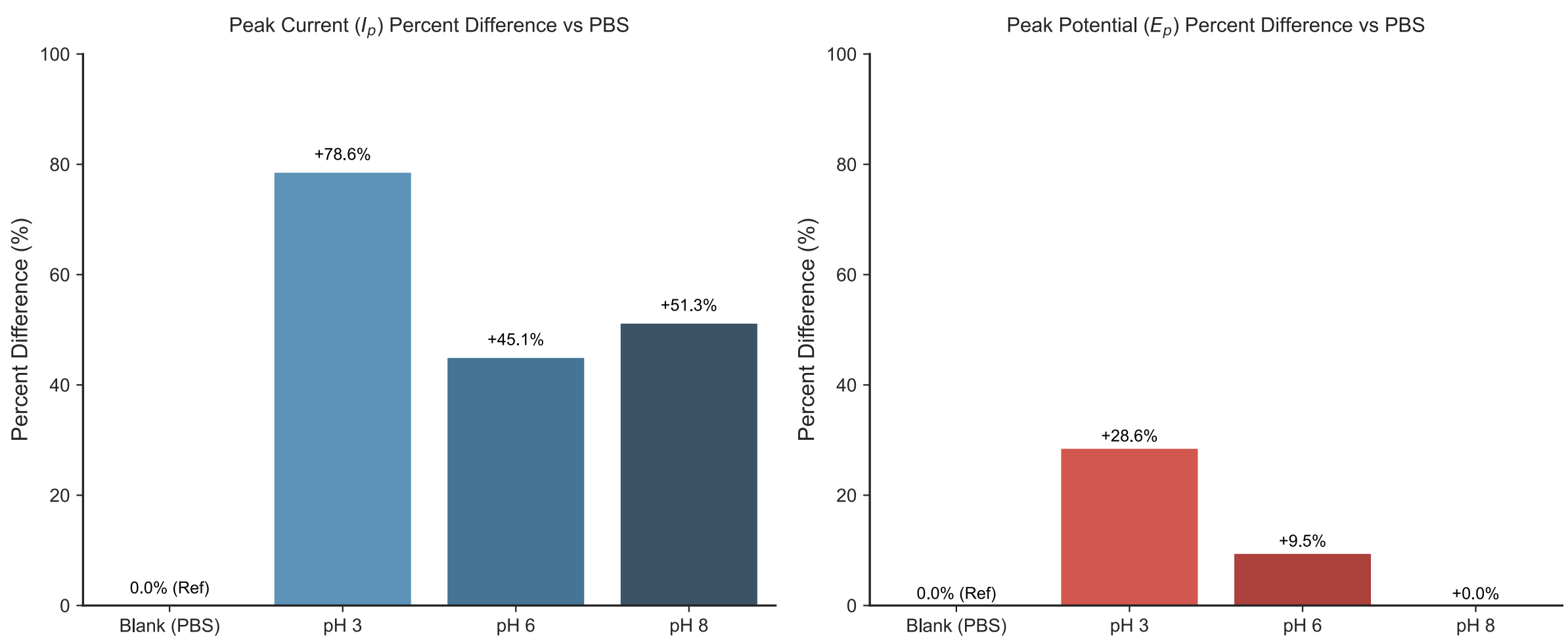

S9: Percentage differences in peak current (I_p, left) and oxidation peak potential (E_p, right) measured across pH 3, 6, and 8 relative to baseline PBS (0.0% Ref). Minimal potential shifts are observed within the physiological window (+9.5% at pH 6 and +0.0% at pH 8), while the mediator retains electroactive response across all conditions (+45.1% I_p at pH 6 and +51.3% I_p at pH 8).

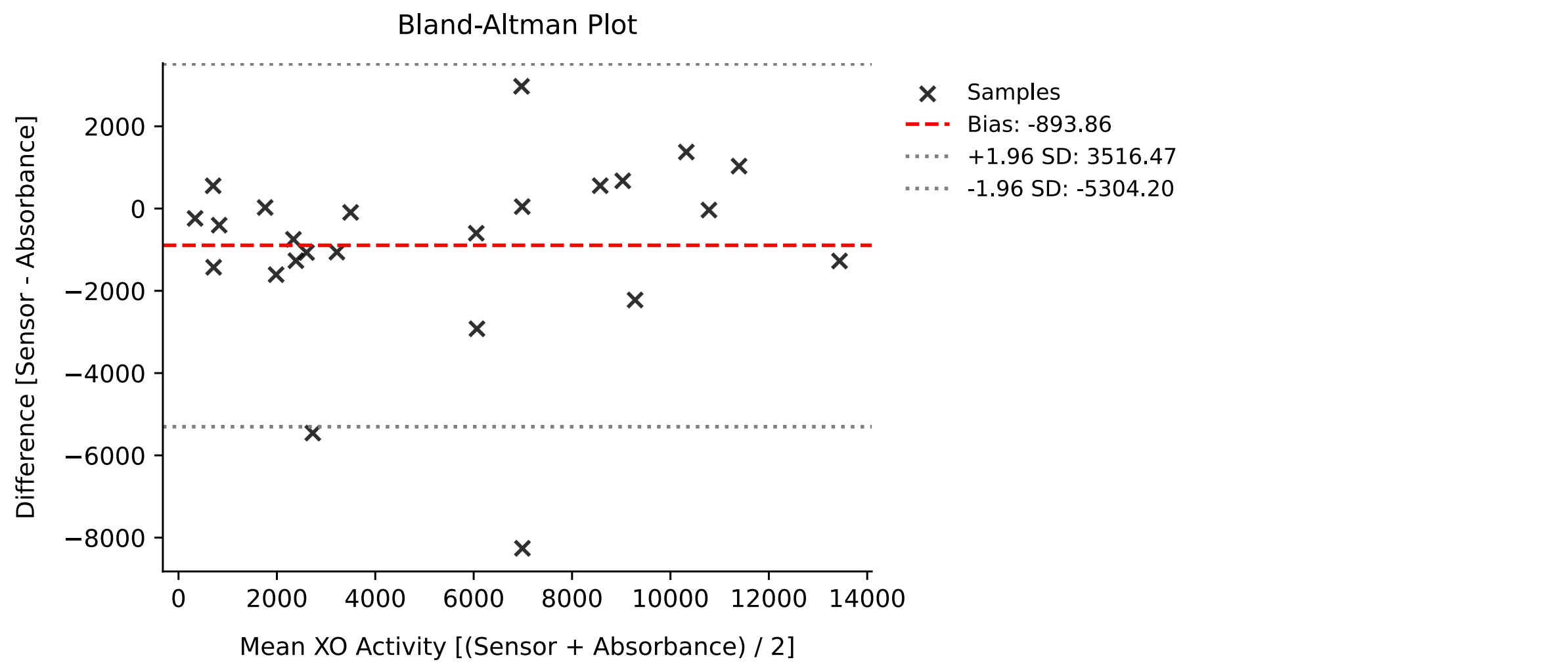

S10: Bland–Altman agreement analysis comparing urinary Xanthine Oxidase (XO) activity quantified by the capillary-fill electrochemical sensor against the commercial colorimetric reference assay across spiked human urine samples. The red dashed line represents the mean systemic bias (-893.86), while the gray dotted lines indicate the upper (+3516.47) and lower (-5304.20) $\mathbf{95}\%$ limits of agreement ($\text{mean} \pm \mathbf{1.96} \times \text{SD}$). Increased variance at higher concentrations reflects proportional error typical of complex urine matrix comparisons between optical and electrochemical sensing modalities.

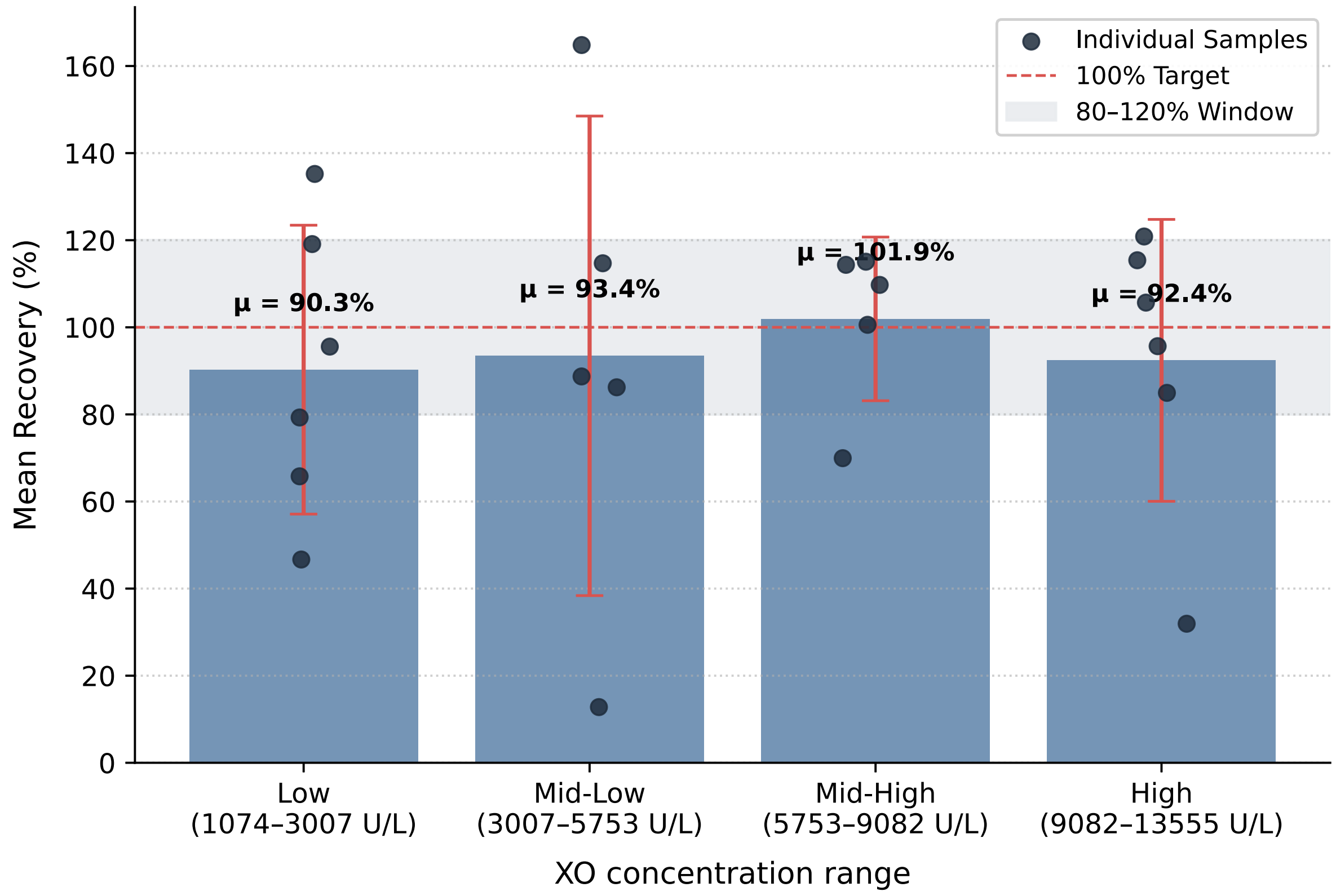

S11:Mean percentage recovery across concentration intervals. Blue bars represent mean recovery (μ), and red vertical error bars represent one standard deviation (± SD). The shaded grey band denotes the window from 80% to 120%, and the red dashed line indicates 100% target recovery. Interval specifications: Low (1074–3007 units: n = 6, %RSD = 36.7%), Mid-Low (3007–5753 units: n = 5, %RSD = 58.9%), Mid-High (5753–9082 units: n = 5, %RSD = 18.4%), High (9082–13555 units: n = 6, %RSD = 35.0%).
